# Advanced Oxidative Protein Products Drive Trophoblast Cells Into Senescence by Inhibiting the Autophagy: The Potential Implication of Preeclampsia

**DOI:** 10.3389/fcell.2022.810282

**Published:** 2022-03-09

**Authors:** Zhengjuan Li, Shuoshi Wang, Liping Li

**Affiliations:** ^1^ Department of Obstetrics, Shenzhen People’s Hospital (The Second Clinical Medical College, Jinan University, The First Affiliated Hospital, Southern University of Science and Technology), Shenzhen, China; ^2^ Department of Obstetrics and Gynecology, The Second Affiliated Hospital, School of Medicine, South China University of Technology, Guangzhou, China

**Keywords:** preeclampsia, trophoblast cells, AOPPs, oxidative Stress, senescence, autophagy, mTOR, p53

## Abstract

**Introduction:** Advanced oxidation protein products (AOPPs), the novel marker of oxidative stress, have been found to be elevated in preeclampsia (PE). To date, the effect of AOPPs on the senescence of trophoblast cells is still unclear. In this study, we investigated whether AOPPs promoted the senescence of trophoblast cells and explored the underlying mechanisms of AOPPs-induced aging process which may facilitate the progression of PE.

**Methods:** The trophoblast cell line HTR-8/SV neo cells were cultured in the presence of PBS, AOPPs, AOPPs plus an anti-oxidant N-acetyl-L-cysteine (NAC). In some experiments, cells were pre-treated with rapamycin (an activator of autophagy), 3-MA (an inhibitor of autophagy), or cyclic pifithrin-α (PFT-α, an antagonist of p53), and then treated with AOPPs. Cellular senescence was analyzed by measuring the levels of senescence-associated β-galactosidase (SA β-Gal), senescence-associated heterochromatin foci (SAHF), mitochondrial membrane potential (ΔΨm), and cell cycle. Cell autophagic flux was analyzed by measuring tandem fluorescence-tagged LC3 reporter (mCherry-EGFP-LC3). Levels of p53, phosphorylated p53 (*p*-p53), p21, BECN1, p62, *p*-mTOR and *p*-p70S6K were measured by western blot.

**Results:** Treatment with AOPPs significantly increased the levels of SA β-Gal and SAHF, the percentage of cells in the G0/G1 phase, and decreased cell ΔΨm compared with the control group. Co-treatment with NAC and AOPPs significantly reversed AOPPs-induced senescence. Pre-treatment with rapamycin or 3-MA significantly inhibited or promoted AOPPs-induced senescence, respectively. In addition, administration of AOPPs significantly decreased the numbers of mCherry^+^EGFP^+^ autophagosomes and mCherry^+^EGFP^-^ autolysosomes in cells compared with cells treated with PBS. Furthermore, AOPPs significantly increased the levels of proteins *p*-p53, p21, *p*-mTOR and *p*-p70S6K compared with the control group. Pre-treatment with rapamycin or PFT-α significantly down-regulated the levels of SA *β*-Gal, SAHF, p-p53, p21, autophagy related protein p62, the percentage of cells in the G0/G1 phase, and significantly up-regulated ΔΨm, autophagy related protein BECN1, autophagosomes and autolysosomes compared with cells only treated with AOPPs.

**Conclusion:** AOPPs may induce trophoblast cell senescence by inhibiting the autophagy process in a p53/mTOR/p70S6K-dependent pathway.

## Introduction

Preeclampsia (PE), a complication associated with 2–8% of pregnancies, is an obstetric syndrome induced during pregnancy that not only affects multiple organs and multiple systems in the mother but also impairs the development of the fetus (Steegers et al., 2010). Substantial evidences have suggested that trophoblast cells play a critical role in placental development (Oreshkova et al., 2012; Staud and Karahoda, 2018). Placental trophoblast cells are the structural and biochemical barrier between maternal and fetal tissues. On one hand, trophoblast cells differentiate into extravillous trophoblast cells invading the maternal spiral artery, where trophoblast cells replace the arterial media and transform the uteroplacental circulation into a high-flow, low-resistance system. On the other hand, trophoblast cells interact with decidual stromal cells, immune cells and other non-immune cells that compose the maternal-fetal interface (Oreshkova et al., 2012). Malfunction of trophoblast cells may not only cause a number of complications, including PE and intrauterine growth restriction, but also lead to fetal programming that increases predisposition to various metabolic and central nerve system diseases later in adult life (e.g., type 2 diabetes mellitus, hypertension, metabolic syndrome, depression, schizophrenia and autism spectrum disorders (Staud and Karahoda, 2018)).

Advanced oxidation protein products (AOPPs) encompass di-tyrosine containing cross-linked protein products, which mostly form after the oxidation of albumin *in-vivo*. Evidences have suggested that the expression of AOPPs is higher in diverse diseases, including diabetes (Heidari et al., 2020), obesity (Fejfer et al., 2017) and polycystic ovary syndrome (Melo et al., 2016). Increasing evidences indicate that women with high plasma levels of AOPPs have a higher risk of developing PE during their pregnancies. Karacay et al. (2010) evaluated concentrations of plasma AOPPs in women, including 27 women with gestational diabetes mellitus (GDM), 27 women with PE and 29 women with noncomplicated singleton pregnancies between 24 and 36 weeks of gestation, and found that AOPPs plasma expression significantly elevated in those with PE or GDM. Huang et al. (2013) evaluated concentrations of plasma AOPPs in 22 women with mild PE and 28 women with severe PE. Their data showed that levels of plasma AOPPs arose with the progression of PE, and demonstrated a positive relation between concentrations of plasma AOPPs and 24 h proteinuria excretion. However, Noyan et al. (2006) found that levels of AOPPs in the plasm of women with PE and eclampsia did not have significant differences with normal pregnancy women. Therefore, the role of AOPPs in PE remains to be illuminated.

In addition, Komosinska-Vassev et al. (2012) found that concentrations of AOPPs significantly elevated in 177 healthy elderly people compared with young subjects. It has been demonstrated that AOPPs play an important role in vascular endothelia aging (Luna et al., 2016) and aging-associated bone loss (Melough et al., 2017). As a crucial feature of mammalian cells, senescence can be both protective and deathful. Oncogene-triggered senescence can suppress tumor growth (Calcinotto et al., 2019), whereas cell aging may be responsible for atherosclerosis (Wang and Bennett, 2012) and neurodegeneration (Hou et al., 2019). Senescent cells produce a complex secretome, the senescence-associated secretory phenotype (SASP), which can be detrimental in tissue repairing and organ aging (Birch and Gil, 2020). Emerging data have shown that placental oxidative stress is a critical intermediary event in the pathology of PE and intrauterine growth restriction (Hsieh et al., 2012; Zsengellér et al., 2016). Meanwhile, it has been demonstrated that placental senescence might be critical in the pathogenesis of PE (Biron-Shental et al., 2010; Cindrova-Davies et al., 2018). Smith et al. (2013) have hypothesized that oxidative stress causes changes in placental proteins, lipids and DNA, which may induce a form of advanced aging, leads to placental insufficiency and inability to meet the demands of the growing fetus, and ultimately causes fetal demise. Mayne et al. (2017) have analyzed DNA methylation data from publication data and proved that placentas from early-onset PE pregnancies present accelerated aging.

Autophagy is a highly orchestrated process involved in the degradation of damaged proteins and organelles inside the cell, and plays an essential role in maintaining genome homeostasis (Dikic and Elazar, 2018). As an evolutionarily conserved catabolic process, autophagy modulates various physiological and pathophysiological situations, including starvation adaptation, clearance of damaged proteins and organelles, development, elimination of pathogens, cell survival and death, tumor suppression, and antigen presentation (Klionsky et al., 2016). Disorder of autophagy causes many diseases, including neurodegenerative diseases (Park et al., 2020) and some metabolic diseases, including insulin resistance, diabetes mellitus, obesity, atherosclerosis and osteoporosis (Kim and Lee, 2014). However, Bernard et al. (Bernard et al., 2020) have shown that in serum starvation, fibroblasts are driven into senescence by sustained autophagy induced by oxidative stress, which can be reversed by inhibiting autophagy. Therefore, our study aimed to investigate the relationship between placental oxidative stress, trophoblast cell senescence, autophagy and the pathogenesis of PE. We found that AOPPs induced trophoblast cell senescence through oxidative stress, and inhibited autophagy process through activating p53 following activating mTOR and its downstream molecule p70S6K.

## Materials and Methods

### Preparation of AOPPs

AOPPs were prepared as described previously (Witko-Sarsat et al., 1998). Briefly, Bovine serum albumin (BSA, Solarbio, Beijing, China) was exposed to hypochlorous acid (HOCl, Sangon Biotech, Shanghai, China) for 30 min and dialyzed overnight against PBS (Hyclone, South Logan, United States) to remove free HOCl. Concentrations of AOPPs in the preparation were determined using a microplate reader (Model MR 5000, Dynatech, France). The concentrations of BSA were 72.4 ± 9.8 nmol/mg protein in prepared AOPPs-BSA (AOPPs) and 0.2 ± 0.02 nmol/mg protein in PBS-BSA (PBS).

### Senescence-Associated β-Galactosidase Stain

To measure cellular senescence, HTR-8/SV neo cells (Shanghai Biological Technology, Shanghai, China), the first-trimester extra-villous trophoblast cell line, were stained with the senescence-associated β-galactosidase (SA β-Gal, a widely accepted senescence marker) (Beyotime Institute of Biotechnology, Nanjing, China) according to the manufacturer’s instruction. In brief, cells were seeded in 6-well plates for 24 h, and then pre-treated with rapamycin (500 nM, Sigma, St. Louis, MO, United States) or 3-MA (2 mM, Sigma) for 1 h, or pre-treated with cyclic pifithrin-α (PFT-α) (30 mM, MCE, New Jersey, United States) for 6 h, and then treated with AOPPs (200 μg/ml) for 48 h. In selected experiments, cells were treated with PBS, AOPPs (200 μg/ml), or AOPPs (200 μg/ml) plus N-acetyl-L-cysteine (NAC) (1 mM, Sigma) for 48 h. Images containing more than 200 cells per high magnification field of view were randomly taken by fluorescent microscope Leica DMI4000B (Leica, Wetzlar, Germany). The numbers of total and blue cells were counted using Image J software (National Institutes of Health, Bethesda, MD, United States). The percentage of SA β-Gal^+^ cells was calculated as the ratio of the number of blue cells to the number of total cells. The assay was carried out in triplicate and repeated six times independently.

### Evaluation of Mitochondrial Membrane Potential (ΔΨm)

HTR-8/SV neo cells were seeded in 12-well plates and cultured for 24 h, and then treated with PBS, AOPPs (200 μg/ml), and AOPPs (200 μg/ml) plus NAC (1 mM). In selected experiments, cells were pre-treated with rapamycin (500 nM) or 3-MA (2 mM) for 1 h, or PFT-α (30 mM) for 6 h, and then treated with AOPPs (200 μg/ml) for 48 h. Evaluation of ΔΨm was conducted using a lipophilic probe 5,5′,6,6′-tetrachloro-1,1′,3,3′-tetraethyl-benzimidazolylcarbocyanine iodide (JC-1, Sigma), which is able to selectively penetrate mitochondria. In healthy cells with high ΔΨm, JC-1 forms complexes known as J-aggregates, while in unhealthy cells with low ΔΨm, JC-1 remains as the monomeric form. When excited at 510 nm, JC-1 monomers emit a green fluorescence with a maximum at 527 nm. Aggregates of JC-1 emit an orange-red fluorescence with a maximum at 590 nm. A total of 3 × 10^5^ cells were added 0.5 mg/ml JC-1, and incubated for 30 min at 37°C in the dark. Cells were then washed and analyzed by a Beckman flow cytometer (Beckman Coulter, CA, United States). Data was analyzed using FlowJo software (Ashland, OR, United States).

### Immunostaining and Confocal Fluorescence Microscopy Assay

HTR-8/SV neo cells were seeded in 96-well plates and cultured for 24 h, and then treated with PBS, AOPPs (200 μg/ml), or AOPPs (200 μg/ml) plus NAC (1 mM) for 48 h. In selected experiments, cells were pre-treated with rapamycin (500 nM) or 3-MA (2 mM) for 1 h, or PFT-α (30 mM) for 6 h, and then treated with AOPPs (200 μg/ml) for 48 h. The formation of senescence-associated heterochromatin foci (SAHF), a senescence marker, can be visualized as the punctate nuclear focus when cells were stained with DAPI (Biolegend, San Diego, CA, United States) and the Ab against K9 tri-methylated histone 3 (K9M-H3, Abcam, Cambridge, MA, United States). For confocal fluorescence analysis, cells were fixed in 4% paraformaldehyde for 20 min at room temperature, and blocked in PBS containing 5% BSA and 0.1% Triton X-100 (all from Sangon Biotech, Shanghai, China). Fixed cells were washed and incubated with rabbit anti-K9M-H3 Ab at 4°C overnight, and then washed and incubated with Alexa Flour 488-conjugated anti-rabbit secondary Ab (Thermo Fisher Scientific, Franklin, MA, United States) at room temperature for 1 h. After washing with PBS, cells were stained with DAPI for 5 min. Samples were observed under the Leica confocal microscopy TCS SP5 (Leica) and analyzed using LCS Leica confocal software (Leica). Cells with condensed imaging of K9M-H3 co-localized with DAPI in the nuclei were considered as SAHF^+^ cells. Percentages of SAHF^+^ cells were calculated.

### Cell Transfection with mCherry-EGFP-LC3

To directly monitor autophagy flux, a fluorescence assay, tandem monomeric mCherry-EGFP-tagged LC3 was employed. HTR-8/SV neo cells were cultured in 24-well plates for 24 h to reach around 70% confluence. Cells were transfected with a lentivirus-mediated mCherry-EGFP-LC3 (Hanbio company, Shanghai, China) to generate mCherry-EGFP-LC3-expressing cells. According to the manufacturer’s instructions, 250 µl cell culture media containing 2.5 × 10^5^ transducing units of lentivirus was added to the plate. After 4 h of culture, another 250 µl culture media was added, and cells were cultured for 20 h. Subsequently, cells were treated with rapamycin (500 nM) or 3-MA (2 mM) for 1 h, or treated with PFT-α (30 mM) for 6 h, and then treated with AOPPs for 48 h. Cells only treated with AOPPs served as positive controls. Cells only treated with PBS served as negative controls. Cell expression of mCherry (red) or EGFP (green) was observed under the Leica confocal microscopy TCS SP5 (Leica) and analyzed using LCS Leica confocal software (Leica). The assay was carried out in triplicate and repeated six times independently.

### Analysis of the Cell Cycle

HTR-8/SV neo cells were seeded in 12-well plates and cultured for 24 h. Cells were then treated with PBS, AOPPs (200 μg/ml), AOPPs (200 μg/ml) plus NAC (1 mM) and cultured for 48 h. In selected experiments, cells were pre-treated with rapamycin (500 nM) or 3-MA (2 mM) for 1 h, or PFT-α (30 mM) for 6 h, and then treated with AOPPs (200 μg/ml) for 48 h. The cell cycle was measured by DNA fragment staining with propidium Iodide (PI, Jiangsu Keygen Biotech, Nanjing, China). According to the manufacturer’s instructions, cells (5 × 10^5^) were washed and fixed with 70% cold ethanol for 2 h at 4 °C. After washing twice with cold PBS, cells were stained with 500 μl working solution containing 450 μl PI solution and 50 μl RNase for 30 min at room temperature. Cells were washed, and the fluorescence intensities of viable cells were measured using a Beckman flow cytometer (Beckman Coulter). Data were analyzed using FlowJo software.

### Western Blotting

HTR-8/SV neo cells were seeded in 6-well plates and cultured for 24 h, and then treated with PBS, AOPPs (200 μg/ml), AOPPs (200 μg/ml) plus NAC (1 mM) for 48 h. In selected experiments, cells were pre-treated with rapamycin (500 nM) or 3-MA (2 mM) for 1 h, or PFT-α (30 mM) for 6 h, then treated with AOPPs for 48 h. Cells were subsequently lysed in the lysis buffer (Beyotime Institute of Biotechnology) for 10 min on ice. Cells were centrifugated at 13,000 g for 10 min at 4°C, and the cell lysates were collected. Protein concentrations were determined using the Pierce^®^ BCA protein assay kit (Thermo Fisher Scientific). Approximately 30 μg of protein were separated on the 4–12% Bis–Tris Gel (Genscript, Nanjing, China) and transferred to the PVDF membrane (Millipore, Billerica, MA, United States). The membrane was incubated with primary Abs against p53, p21 (both from Santa Cruz, California, United States), phosphorylated p53 (Ser15) (*p*-p53), p62, BECN1, p-mTOR (Ser2448), *p*-p70S6K (Thr389) and β-Actin (all from Cell Signaling Technology, Beverly, MA, United States) overnight at 4°C. The membrane was then washed and incubated with horseradish peroxidase (HRP)-coupled goat-anti-mouse or goat-anti-rabbit secondary Ab IgG (H + L) (Thermo Fisher Scientific). The membrane was washed, and treated with the Pierce^®^ ECL Western Blotting Substrate (Thermo Fisher Scientific) for color exposure. Image J software was used to detect the gray value of the strip. The expression of *p*-p53 was analyzed semi-quantitatively using p53 as the internal reference protein. The expression of target proteins p53, p21, p62, BECN1, *p*-mTOR and *p*-p70S6K was analyzed semi-quantitatively using *β*-Actin as the internal reference protein.

### Statistical Analysis

All statistical analyses were performed using Graphpad Prism eight software (GraphPad, Bethesda, MD, United States). Data were analyzed using one-way ANOVA with Bonferroni test. Results were presented as means ± SDs. A *p* value of <0.05 was considered significant among analyzed groups.

## Results

### AOPPs Induced Cell Senescence in an Oxidation-Dependent Manner

**FIGURE 1:**
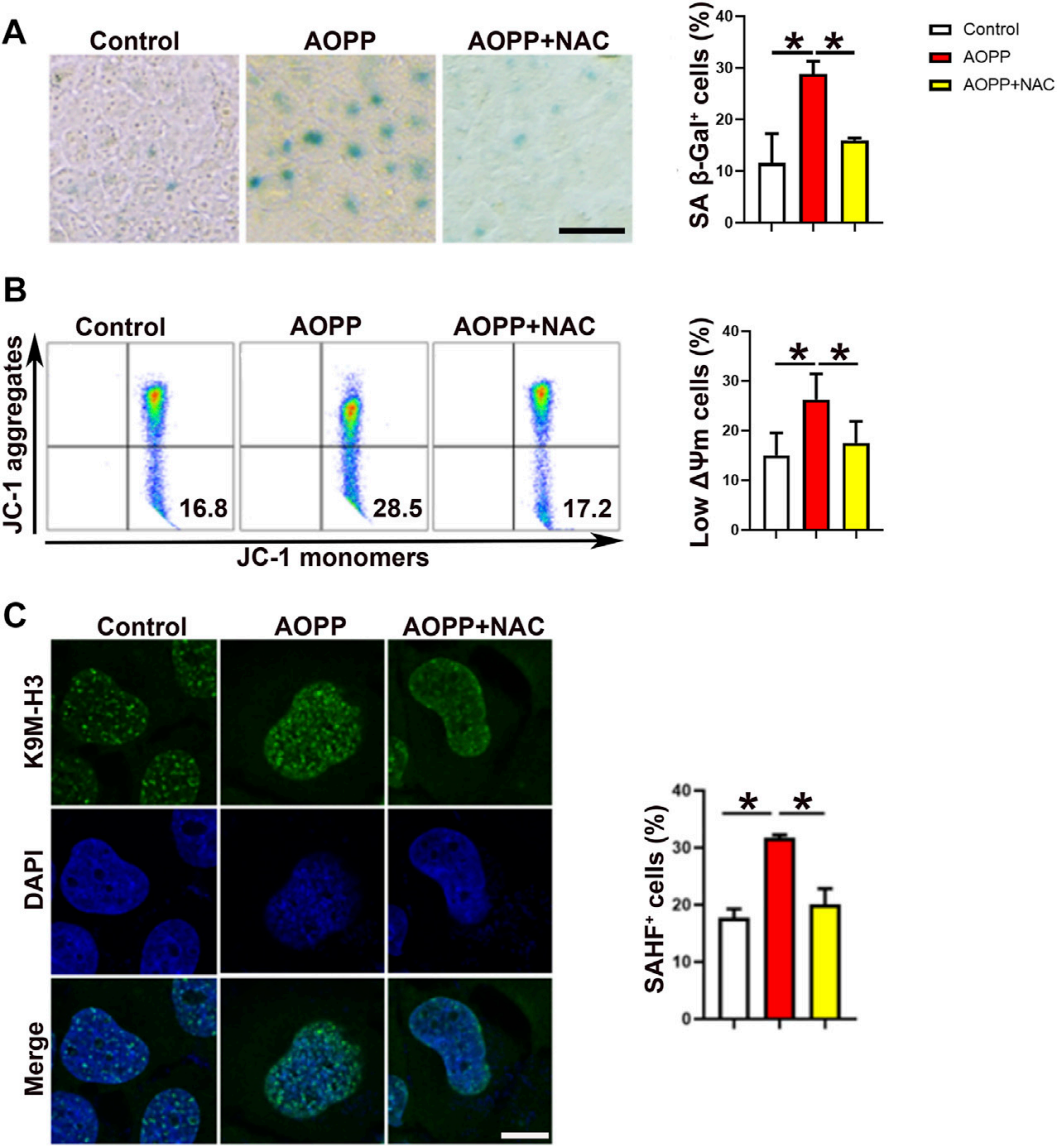
AOPPs induce cell senescence in an oxidation-dependent manner. HTR-8/SV neo cells were treated with PBS, AOPPs, or AOPPs plus NAC, and cultured for 48 h. **(A)** Representative images of SA β-Gal staining (left), and the percentages of SA β-Gal^+^ cells were presented (right). Scale bar: 50 μm. **(B)** Representative flow cytometry scatter plots of JC-1 staining were shown, and the cells in the right lower quadrant represented cells with low ΔΨm (left). The histograms represented the percentages of cells with low ΔΨm (right). **(C)** Immunostaining of K9M-H3 (green) and DAPI (blue) and the representative images of SAHF^+^ cells (left), and the percentages of SAHF^+^ cells (right) were presented. Scale bar: 20 μm ^*^ *p* <0.01.

As shown in Figure 1A, treatment with AOPPs markedly increased the expression of SA *β*-Gal on HTR-8/SV neo cells compared with the control group (*p* <0.01), while co-treatment with AOPPs and NAC, an anti-oxidant, significantly suppressed the effect of AOPPs, demonstrating the role of oxidative stress in AOPPs-induced cell senescence. Mitochondrial membrane potential (ΔΨm) is a critical indicator of mitochondrial function, and decreased ΔΨm is closely associated with cell senescence (Mansell et al., 2021). AOPPs significantly decreased the ΔΨm of HTR-8/SV neo cells, while co-treatment with AOPPs and NAC significantly increased cell ΔΨm compared with cells treated with AOPPs **(** *p* <0.01, Figure 1B). In addition, as shown in Figure 1C, AOPPs significantly increased the percentage of SAHF^+^ cells as compared to controls (*p* <0.01), while co-treatment with AOPPs and NAC significantly decreased the percentage of SAHF^+^ cells compared with cells only treated with AOPPs (*p* <0.01).

### Inhibition of Autophagy Promoted the Pro-Senescent Effect of AOPPs

**FIGURE 2:**
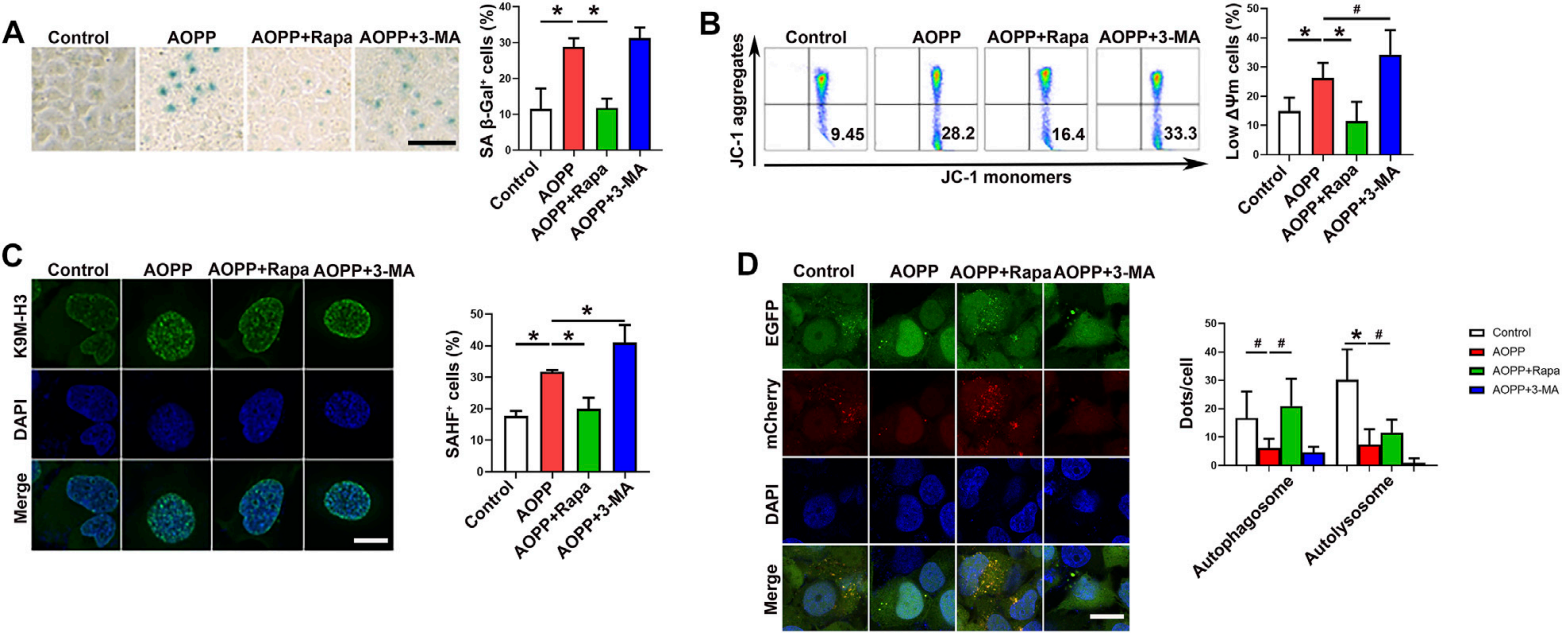
Inhibition of autophagy promoted the pro-senescence effect of AOPPs. Cells were treated with PBS or AOPPs and cultured for 48 h. In selected groups, cells were pre-treated with rapamycin or 3-MA for 1 h, and then treated with AOPPs, and cultured for 48 h. **(A)** Representative images of SA β-Gal staining (left), and the percentages of SA β-Gal^+^ cells were presented (right). Scale bar: 50 μm. **(B)** Representative flow cytometry scatter plots of JC-1 staining were shown, and cells in the right lower quadrant represented cells with low ΔΨm (left). The histograms represented the percentages of cells with low ΔΨm (right). **(C)** Immunostaining of K9M-H3 (green) and DAPI (blue) and the representative images of SAHF^+^ cells (left), and the percentages of SAHF^+^ cells were presented (right). Scale bar: 20 μm. **(D)** Autophagic flux was measured by analyzing tandem fluorescence-tagged LC3 reporter (mCherry-EGFP-LC3). Yellow mCherry^+^EGFP^+^ LC3 puncta represented autophagosomes and red mCherry^+^EGFP^-^ LC3 puncta represented autolysosomes, and the percentages of autophagosomes and autolysosomes were presented (right). Scale bar: 20 μm. Data were presented as means ± SDs of at least six independent experiments. ^#^ *p* <0.05, ^*^ *p* <0.01.

To investigate whether autophagy was associated with the pro-senescent effect of AOPPs, HTR-8/SV neo cells were pre-treated with rapamycin, an activator of autophagy, or 3-MA, an inhibitor of autophagy, before cells were treated with AOPPs. Our results showed that pre-treatment with rapamycin significantly inhibited AOPPs-increased percentages of SA β-Gal^+^ cells (Figure 2A). The expression of SA β-Gal was higher in cells treated with 3-MA and AOPPs compared with cells only treated with AOPPs, but the difference was not significant (Figure 2A). In addition, treatment with rapamycin and AOPPs significantly decreased the percentage of low ΔΨm cells compared with cells only treated with AOPPs (*p* <0.01). While treatment with 3-MA and AOPPs significantly increased the percentage of low ΔΨm cells compared with cells only treated with AOPPs (*p* <0.05, Figure 2B). Moreover, treatment with rapamycin and AOPPs significantly inhibited AOPPs-increased percentages of SAHF^+^ cells (Figure 2C), while treatment with 3-MA and AOPPs significantly promoted AOPPs-increased percentages of SAHF^+^ cells (Figure 2C). Our results suggested that while activating autophagy decreased AOPPs-induced senescence, inhibiting autophagy increased AOPPs-induced senescence.

We next analyzed autophagic flux by measuring tandem fluorescence-tagged LC3 reporter (mCherry-EGFP-LC3) based on different pH stability of fluorescent proteins EGFP and mCherry. EGFP signal is quenched in the acidic environment when LC3-formed autophagosomes are fused with lysosomes for degradation (Klionsky et al., 2016). Yellow mCherry^+^EGFP^+^ LC3 puncta represent autophagosomes and red mCherry^+^EGFP^-^ LC3 puncta represent autolysosomes (Klionsky et al., 2016). As demonstrated in Figure 2D, there were fewer yellow mCherry^+^EGFP^+^ LC3 puncta (autophagosomes) and red mCherry^+^EGFP^-^ LC3 puncta (autolysosomes) in cells treated with AOPPs compared with the control group (*p* <0.05 and *p* <0.01). Treatment with rapamycin and AOPPs significantly increased numbers of yellow autophagosomes and red autolysosomes compared with cells only treated with AOPPs (*p* <0.05 for both comparisons). Our results indicated that AOPPs inhibited the formation of autophagosomes and autolysosomes in HTR-8/SV neo cells.

### Inhibition of p53 Reversed the Pro-Senescence Effect of AOPPs.

**FIGURE 3:**
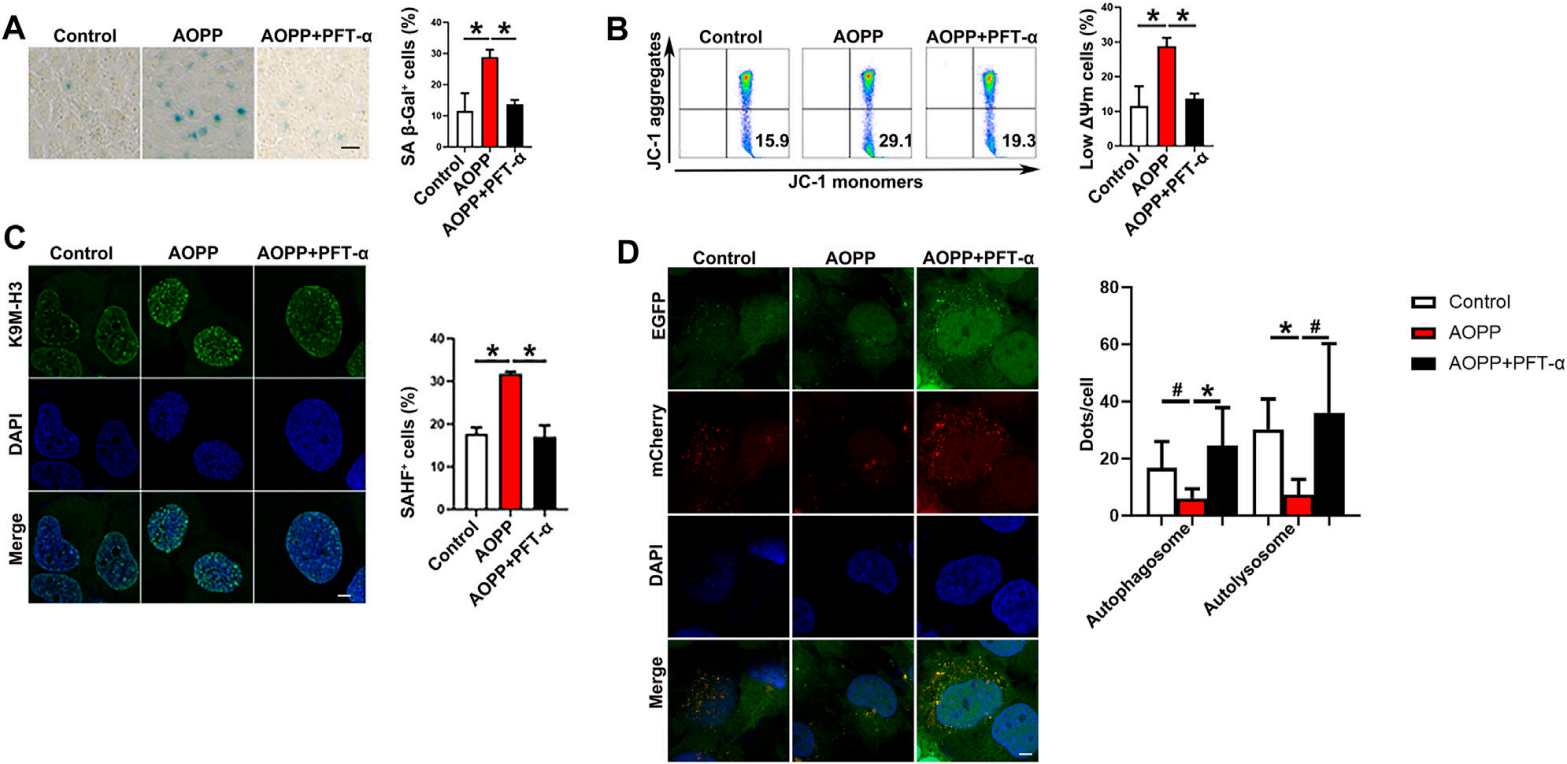
Inhibition of p53 reversed the pro-senescence effect of AOPPs. HTR-8/SV neo ells were treated with PBS or AOPPs and cultured for 48 h. In selected experiments, cells were pre-treated with PFT-α for 6 h, then treated with AOPPs, and cultured for 48 h. **(A)** Representative images of SA β-Gal staining (left), and the percentages of SA *β*-Gal^+^ cells were presented (right). Scale bar: 50 μm. **(B)** Representative flow cytometry scatter plots of JC-1 staining were shown, and cells in the right lower quadrant represented cells with low ΔΨm (left). The histograms represented the percentages of cells with low ΔΨm (right). **(C)** Immunostaining of K9M-H3 (green) and DAPI (blue) and the representative images of SAHF^+^ cells (left), and the percentages of SAHF^+^ cells were presented (right). Scale bar: 20 μm. **(D)** Autophagic flux was measured by analyzing tandem fluorescence-tagged LC3 reporter (mCherry-EGFP-LC3). Yellow mCherry^+^EGFP^+^ LC3 puncta represented autophagosomes and red mCherry^+^EGFP^-^ LC3 puncta represented autolysosomes, and the percentages of autophagosomes and autolysosomes were presented (right). Scale bar: 20 μm. Data were presented as means ± SDs of at least six independent experiments. ^#^ *p* <0.05, ^*^ *p* <0.01.

p53 Functions as a transcription factor involved in cell-cycle control, DNA repair, apoptosis and cellular stress responses (Rufini et al., 2013; Hafner et al., 2019). In addition, p53 activation also modulates cellular senescence and organismal aging (Rufini et al., 2013; Hafner et al., 2019). To confirm the role of p53 in AOPPs-induced senescence, PFT-α, an antagonist of p53 (Leeper et al., 2013), was employed. Pre-treatment with PFT-α significantly down-regulated AOPPs-increased levels of SA β-Gal (Figure 3A). Treatment with PFT-α and AOPPs significantly decreased the percentages of low ΔΨm cells (*p* < 0.01, Figure 3B) and SAHF^+^ cells (*p* < 0.01, Figure 3C) compared with cells only treated with AOPPs. Meanwhile, pre-treatment with PFT-α significantly increased the yellow autophagosomes and red autolysosomes compared with cells only treated with AOPPs (*p* <0.01 and *p* <0.05, Figure 3D). Our data suggested that inhibition of p53 reversed the pro-senescence effect of AOPPs.

### AOPPs Induced the Cell Cycle Arrest of Trophoblast Cells

**FIGURE 4:**
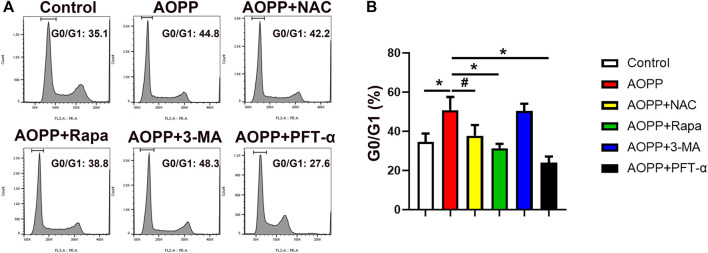
AOPPs treatment induced trophoblast cells cycle arrest. HTR-8/sv neo cells were treated with PBS, AOPPs, or AOPPs plus NAC, and cultured for 48 h. In some experiments, cells were pre-treated with rapamycin or 3-MA for 1 h, or PFT-α for 6 h, and then treated with AOPPs, and cultured for 48 h. Representative flow cytometry scatter plots of PI staining **(A)** were shown, and the percentages of cells in the G0/G1 cell cycle phase were presented. The histograms represented the percentages of cells in the G0/G1 cell cycle phase **(B)**. Data were presented as means ± SDs of at least six independent experiments. ^#^ *p* < 0.05, ^*^ *p* < 0.01.

Senescence is an irreversible cell cycle arrest (Calcinotto et al., 2019). We next examined the cell cycle of AOPPs-treated trophoblast cells. As shown in Figure 4, administration of AOPPs significantly increased the percentage of cells in the G0/G1 phase compared with controls (*p* <0.01). Co-treatment with NAC and AOPPs decreased the percentage of cells in the G0/G1 phase compared with cells only treated with AOPPs (*p* <0.05). In addition, pre-treatment with rapamycin significantly reduced the percentage of cells in the G0/G1 phase compared with cells only treated with AOPPs (*p* <0.01). We also found that AOPPs-induced cell cycle arrest was reversed by pre-treatment with PFT-α. In accordance with AOPPs-induced senescence, AOPPs treatment induced trophoblast cell cycle arrest through oxidative stress and inhibiting autophagy, and in a p53-dependent manner.

### AOPPs-Mediated Senescence was Through the p53/mTOR/p70S6K Pathway

**FIGURE 5:**
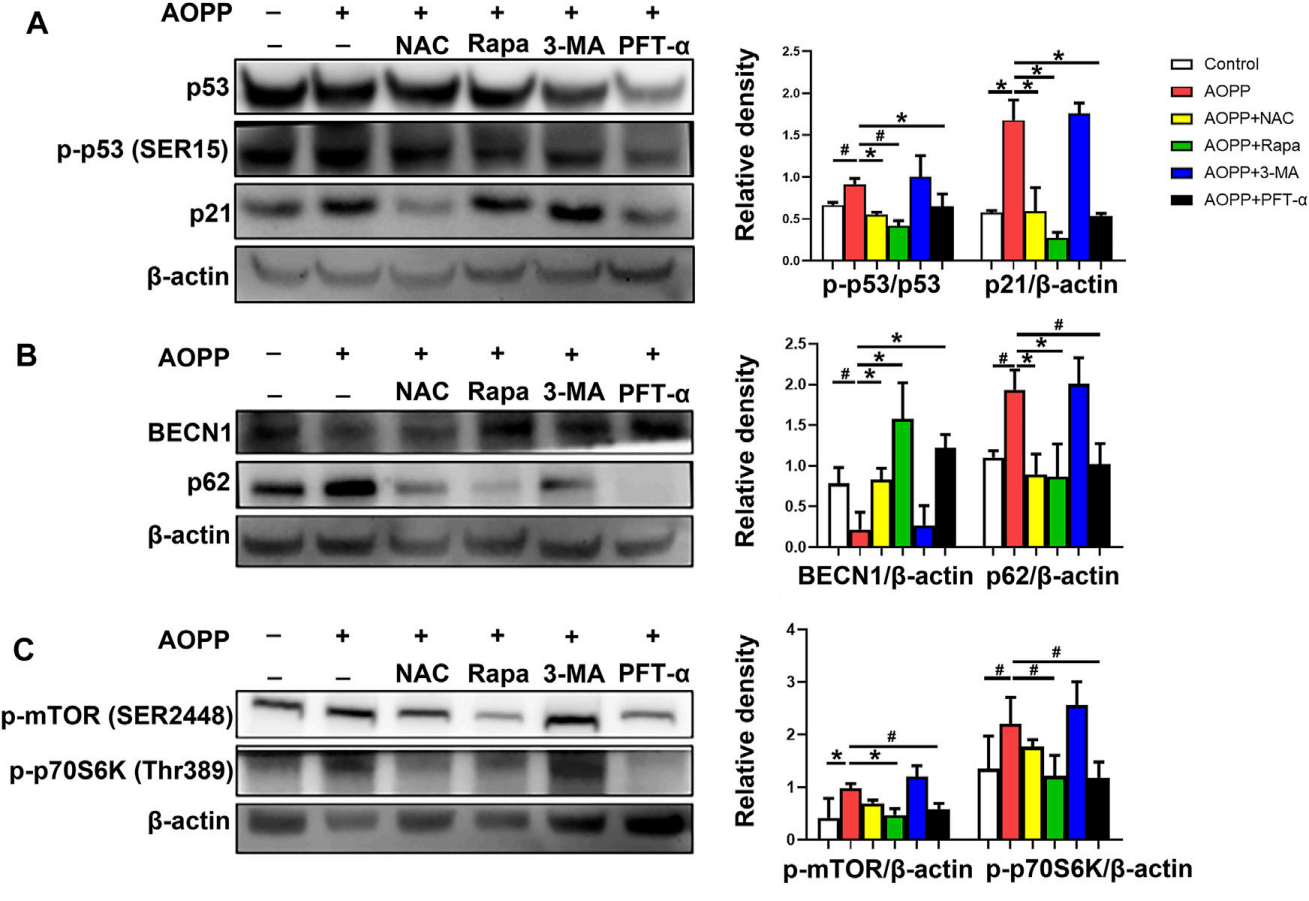
AOPPs-mediated trophoblast cell senescence was though the p53/mTOR/p70S6K pathway. HTR-8/SV neo cells were treated with PBS, AOPPs, or AOPPs plus NAC, and cultured for 48 h. In some selected experiments, cells were pre-treated with rapamycin or 3-MA for 1 h, or PFT-α for 6 h, and then treated with AOPPs, and cultured for 48 h. **(A)** Immune blots of proteins p53, p-p53 and p21 (left), and the levels of *p*-p53 and p21 (right) were presented. **(B)** Immune blots of proteins BECN1 and p62 (left), and the levels of BECN1 and p62 (right) were presented. **(C)** Immune blots of proteins *p*-mTOR and *p*-p70S6K (left), and the levels of *p*-mTOR and *p*-p70S6K (right) were presented. Data were presented as means ± SDs of at least six independent experiments. ^#^ *p* <0.05, ^*^ *p* <0.01.

Since the p53-p21 pathway plays a critical role in senescence induction (Rufini et al., 2013), we explored the p53-p21 pathway in AOPPs-treated HTR-8/SV neo cells. As shown in Figure 5A, AOPPs significantly increased the protein expression level of both *p*-p53 (*p* <0.05) and p21 (*p* <0.01) compared with the control group. Treatment with NAC attenuated the AOPP-increased expression of *p*-p53 and p21 **(** *p* <0.01 for both comparisons). Meanwhile, pre-treatment of rapamycin inhibited the up-regulation of the levels of *p*-p53 (*p* <0.05) and p21 (*p* <0.01) induced by AOPPs. In addition, pre-treatment with PFT-α decreased the levels of *p*-p53 and p21compared with cells only treated with AOPPs (*p* <0.01 for both comparison).

We next examined the expression of proteins BECN1 and p62. BECN1 is a critical component of mammalian autophagy (Yue et al., 2003). The polyubiquitin-binding protein p62 is degraded by autophagy (Pankiv et al., 2007). Therefore, autophagy is accompanied with the up-regulation of BECN1 and down-regulation of p62 (Klionsky et al., 2016). As shown in Figure 5B, treatment with AOPPs significantly decreased the level of BECN1 (*p* <0.05), but increased the level of p62 (*p* <0.05) in HTR-8/SV neo cells compared with controls. However, treatment with NAC and AOPPs significantly increased the expression of BECN1 (*p* <0.01), but decreased the expression of p62 (*p* <0.01) in HTR-8/SV neo cells compared with cells only treated with AOPPs. In addition, cells pre-treated with rapamycin or PFT-α demonstrated a significantly increased level of BECN1 (*p* <0.01 for both comparisons) but decreased level of p62 (*p* <0.01 and *p* <0.05) compared with cells only treated with AOPPs.

We also analyzed the levels of *p*-mTOR and *p*-p70S6K, the p53 pathway downstream molecules. As shown in Figure 5C, AOPPs significantly increased the levels of *p*-mTOR (*p* <0.01) and *p*-p70S6K (*p* <0.05) in HTR-8/SV neo cells compared with controls. In contrast, cells pre-treated with rapamycin significantly decreased the levels of *p*-mTOR (*p* <0.01) and *p*-p70S6K (*p* <0.05) compared with cells only treated with AOPPs. Moreover, pre-treatment with PFT-α significantly decreased the levels of *p*-mTOR and *p*-p70S6K compared with cells only treated with AOPPs (*p* <0.05 for both comparisons). Our results indicated that AOPPs-induced HTR-8/SV neo cell senescence may through the p53/mTOR/p70S6K pathway.

## Discussion

PE is a serious complication that compromises maternal wellbeing and fetal development (Steegers et al., 2010). It has been demonstrated that AOPPs accumulate in the plasm of PE pregnancies (Karacay et al., 2010; Huang et al., 2013). In addition, it has been proved that AOPPs induce trophoblast cell apoptosis by enhancing the oxidative pathway (Wang et al., 2015) and inhibiting the anti-oxidative pathway (Chen et al., 2021). Nevertheless, the relationship between AOPPs and senescence remains unclear.

Increased oxidative stress induces the activation of autophagy to clear damaged proteins and organelles and reverses the activation of p-ATM and gamma H2AX induced by the damage of DNA (Guo et al., 2020). If oxidative stress is persistent, DNA damage will be irreversible and then cell senescence and organ malfunction will be induced (Zhou et al., 2021). In our prior study, we have found that treatment with AOPPs significantly increase the level of reactive oxygen species (ROS) in HTR8/SV neo cells (Wang et al., 2015). In the present study, administration of AOPPs induced elevated senescence markers, including SA β-gal, p53-p21 pathway, SAHF, ΔΨm and cell cycle arrest in trophoblast cells. We speculate that AOPPs may play an important role in trophoblast cell senescence.

Autophagy, a self-eating cellular process, degrades old or unused macromolecules for energy production or recycling within cells (Mizushima, 2007). It is widely known that BECN1, LC3 and p62 are three commonly used indicators for detecting autophagy (Klionsky et al., 2016). When the autophagy pathway is activated, the levels of BECN1 and LC3 increase, and the level of p62 that binds to LC3 decreases. In our study, treatment with AOPPs down-regulated the expression of autophagy-associated proteins of trophoblast cells. However, pre-treatment with rapamycin, an activator of autophagy, attenuated AOPPs-induced senescence, suggesting that AOPPs may induce trophoblast cell senescence through the inhibition of cell autophagy.

Similarly, Nakashima et al. (2020) reported that in early- and late-onset PE pregnancies, a master transcriptional regulator of lysosomal biogenesis the transcription factor EB (TFEB) and its down-stream proteins LAMP1, LAMP2 and cathepsin D decreased compared with normal pregnancies, indicating impaired autophagy in PE pregnancies. Lee et al. (2021) explored the consequences of autophagic deficit in the uterus using tissue-specific *atg7* knockout mice, and found aberrations in vascular permeability, increased accumulation of specific vascular factors and defective endothelial junctions in the uterus. Their results have demonstrated that autophagy in the uterine vessel microenvironment is critical for controlling vascular permeability in murine uterus. In addition, it has been repored that AOPPs impair autophagic flux in macrophage by inducing lysosomal dysfunction through the PI3K-Akt-mTOR pathway, and play an important role in Crohn’s disease progression (Liao et al., 2021). Activated autophagy has been reported in the placentae of women with PE (Nakashima et al., 2019). The conflicting results of autophagy in the placentae of preeclamptic women remains unclear and need to be explored.

mTOR has been proved to play a key role as the main gateway to autophagy (Rabanal-Ruiz et al., 2017). Zhang et al. (Zhang et al., 2018) have proved that AOPPs inhibit autophagy in renal tubular epithelial cells via activation of the PI3K/AKT/mTOR pathway. In addition, Feng et al. (Feng et al., 2005) have reported that p53 and mTOR signaling machineries can cross-talk and coordinately regulate cell growth, proliferation and death. To further investigate the underlying relationship between p53 and mTOR in AOPPs-induced pro-aging effect, we examined the levels of p-mTOR, and its down-stream molecule p-p70S6K protein in HTR-8/SV neo cells treated with AOPPs and PFT-α. We found that AOPPs treatment activated mTOR and its downstream molecular p70S6K, which can be reversed by PFT-α. Our results demonstrated that AOPPs inhibits autophagy in a p53/mTOR/p70S6K-dependent manner. Likewise, it has been reported that autophagy-deficient human extravillous trophoblast cells exhibit poor nuclear translocation of TFEB, reduced expression and defected function of lysosomal proteins, and increased activity of mechanistic target of rapamycin complex 1 (MTORC1) (Nakashima et al., 2020).

In summary, AOPPs may induce trophoblast cell senescence through inhibiting autophagy in a p53/mTOR/p70S6K-dependent manner. Our findings revealed a novel mechanism underlying AOPPs-induced trophoblast cell senescence, which may play a role in the pathogenesis of PE.

## Data Availability

The original contributions presented in the study are included in the article/Supplementary Material, further inquiries can be directed to the corresponding author.
